# Impact of Desert Dust Events on the Cardiovascular Disease: A Systematic Review and Meta-Analysis

**DOI:** 10.3390/jcm10040727

**Published:** 2021-02-12

**Authors:** Alberto Domínguez-Rodríguez, Néstor Báez-Ferrer, Pedro Abreu-González, Sergio Rodríguez, Rocío Díaz, Pablo Avanzas, Daniel Hernández-Vaquero

**Affiliations:** 1 Servicio de Cardiología, Hospital Universitario de Canarias, 38010 Tenerife, Spain; nestor.baez@hotmail.com; 2 Departamento de Enfermería, Universidad de La Laguna, 38200 Tenerife, Spain; 3 CIBER de Enfermedades CardioVasculares (CIBERCV), 28029 Madrid, Spain; 4 Departamento de Fisiología, Facultad de Medicina, Universidad de La Laguna, 38200 Tenerife, Spain; pabreugonzalez@gmail.com; 5 Estación Experimental de Zonas Áridas, EEZA, CSIC, 04120 Almería, Spain; sergio.rodriguez@csic.es; 6 Instituto de Productos Naturales y Agrobiología, IPNA, CSIC, 38206 Tenerife, Spain; 7 Área del Corazón, Hospital Universitario Central de Asturias, 33011 Oviedo, Spain; diazmendezro@gmail.com (R.D.); avanzas@gmail.com (P.A.); dhvaquero@gmail.com (D.H.-V.); 8 Instituto de Investigación Sanitaria del Principado de Asturias, 33011 Oviedo, Spain; 9 Departamento de Medicina, Universidad de Oviedo, 33003 Asturias, Spain

**Keywords:** meta-analysis, cardiovascular mortality, acute coronary syndrome, heart failure, particulate matter, dust, desert dust

## Abstract

Background: Whether or not inhalation of airborne desert dust has adverse health effects is unknown. The present study, based on a systematic review and meta-analysis, was carried out to assess the influence desert dust on cardiovascular mortality, acute coronary syndrome, and heart failure. Methods: A systematic search was made in PubMed and Embase databases for studies published before March 2020. Studies based on daily measurements of desert dust were identified. The meta-analysis evaluated the impact of desert dust on cardiovascular events the same day (lag 0) of the exposure and during several days after the exposure (lags 1 to 5). The combined impact of several days of exposure was also evaluated. The incidence rate ratio (IRR) with 95% confidence intervals (CI) was calculated using the inverse variance random effects method. Results: Of the 589 identified titles, a total of 15 studies were selected. The impact of desert dust on the incidence of cardiovascular mortality was statistically significant (IRR = 1.018 (95%CI 1.008–1.027); *p* < 0.001) in lag 0 of the dust episode, in the following day (lag 1) (IRR = 1.005 (95%CI 1.001–1.009); *p* = 0.022), and during both days combined (lag 0–1) (IRR = 1.015 (95%CI 1.003–1.028); *p* = 0.014). Conclusions: The inhalation to desert dust results in a 2% increase (for every 10 µg/m^3^) in cardiovascular mortality risk.

## 1. Introduction

According to the World Health Organization (Geneva, Switzerland), mitigation of the adverse health effects of exposure to airborne pollutants has become a worldwide health concern [1]. Special attention has focused on pollution related to combustion sources (motor vehicles, industry, biomass burning, etc.), which is composed of a mixture of reactive gases (CO, NOx, and SO_2_, among others) and particulate matter (PM) (fundamentally hydrocarbons, organic material, soot, sulfate, nitrate, etc.). Exposure to such PM plays a key role in cardiovascular disorders attributed to air pollution [2]. The World Health Organization (Geneva, Switzerland) recommends that the population should not be exposed to concentrations of breathable PM with an aerodynamic diameter <10 µm, i.e., PM_10_, of over 50 µg/m^3^.

**Figure 1:**
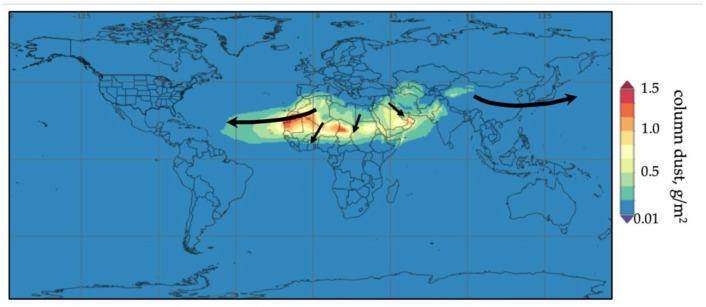
Global mean summer distribution of atmospheric dust according to MERRA-2model. The black arrows indicate the main dust transport routes.

At present, part of the scientific community is investigating the health consequences of the inhalation of airborne desert dust as a component of PM_10_ breathable particles, which affects over 2000 million people [3]. The main dust-emitting regions are found in the so-called Dust Belt, which extends through North Africa, the Middle East, and Inner Asia (Figure 1) [4].

When the dusty air reaches an urban area, the population becomes exposed to the mixture of desert dust and local urban and regional pollution, i.e., PM_10_ is the sum of PM_10_-dust + PM_10_-non-dust (particles generated by industry, motor vehicles, combustion, etc.) [5,6,7,8]. When North African dust reaches southern Europe, PM_10_-dust is usually present in concentrations of between 10–60 µg/m^3^ and represents 25–50% of PM_10_ [6]. However, in North Africa and the Middle East, the PM_10_-dust concentrations can exceed 1000 µg/m^3^ and represent >99% of PM_10_ [9].

Many studies have been conducted in different parts of the world analyzing the possible impact of desert dust on health. However, the available evidence is not fully conclusive. Whereas some studies have reported a small yet significant influence, others have found no impact [3,10,11,12]. The statistical power of time series studies that analyze the influence of dust on health is a very common and underestimated problem. Simulations underscore that a time series study with an inclusion period of four years and an average of 22 events a day would have a statistical power of only 52% [13]. In this scenario, the absence of a statistically significant association in such research studies does not mean that there is no association [14]. For this reason, an analysis is needed comprising a large number of events and which allows us to know the real epidemiological association between desert dust and cardiovascular events. The present study, based on a systematic review and meta-analysis, was carried out to assess the influence of exposure to desert dust on the incidence of cardiovascular mortality, acute coronary syndrome (ACS), and heart failure.

## 2. Materials and Methods

The protocol was registered on PROSPERO (CRD42020181532). We conducted and report this systematic review in accordance with the Preferred Reporting Items for Systematic Reviews and Meta-analyses statement [15]. Our research was carried out according to The Code of Ethics of the World Medical Association, Ferney-Voltaire, France, (Declaration of Helsinki). Patient consent was not required.

### 2.1. Search Strategy and Selection of Studies

A systematic search was made of the PubMed and Embase databases for articles published until March 2020, with the purpose of identifying observational studies including any human population residing in urban or rural areas exposed to desert dust. The search made use of free text and keywords, considering different definitions related to desert dust, sandstorms, and cardiovascular events. The full search strategy is described in the Supplementary Materials Figure S1.

### 2.2. Definition of Exposure to Desert Dust and Study Selection Criteria

We selected studies in which desert dust (tracked in terms of increases in PM_10_ due to desert dust blowing in ambient air [1,16]) was characterized. The inclusion criteria were:

- Comparison of exposure to desert dust versus no such exposure.
- Description of the influence of desert dust (per unit increase) on the incidence of cardiovascular mortality, ACS, or heart failure.
- Identification of the exposure of desert dust in the breathable fraction of the particulate material PM_10_ [16].
- Human studies.
- Studies with a methodologically adequate design. In other words, studies controlling the main confounding factors (meteorological variables and pollutants in gaseous phase) and comprising time series or adopting a cross-case design [17].
- Studies in Spanish or English.

Some Asian studies used total PM as proxy of inhalable dust concentration. A significant difference of this proxy to dust in PM_10_ is not expected due to super-coarse particles (larger than 10 microns) tend to experience quick dry deposition during the transport across Asia.

### 2.3. Objectives

The objective of this study was to evaluate the impact of desert dust on 3 types of cardiovascular events: Cardiovascular mortality, ACS, and heart failure. For this purpose, we examined whether the dust episodes were associated to the cardiovascular event on the same day as the dust episode (lag 0) or whether there was a lag of 1, 2, 3, 4, or 5 days in the cardiovascular event with respect to the dust episode (i.e., lag 1, lag 2, lag 3, lag 4, or lag 5, respectively). The combined impact of several days of exposure was also evaluated 0–1 days (lag 0–1), 2–5 days (lag 2–5), and 0–5 days (lag 0–5) after the dust episode.

### 2.4. Data Extraction and Evaluation of the Quality of the Studies

Three members of the team (A.D.-R., N.B.-F., and P.A.-G.) independently examined the title and abstract of the articles, selecting those publications that met the inclusion criteria. Two other members (S.R. and R.D.) evaluated the previously selected full-text articles and decided their final inclusion on an independent basis. Disagreements were resolved through debate among all the team members. The mentioned evaluating members were blinded to the names of the authors, the institutions, and the names of the journals. Another 3 investigators (A.D.-R., P.A., and D.H.-V.) used the Cochrane ROBINS scale to evaluate the quality of the observational studies [18].

The data extracted from the selected studies were entered in an Excel spreadsheet. If a given study presented various cardiovascular events (cardiovascular mortality, ACS, or heart failure), each result was registered individually. Likewise, if a given study reported several estimated effects (different lags) for each cardiovascular event, these were also compiled. Data extraction included details on citations (first author, title, journal, and date of publication), study design and statistical analysis, place of study (region/country), study period, outcome assessment (cardiovascular mortality, ACS, or heart failure), confounding variables, number of events and estimated effect based on the odds ratio, relative risk, or incidence rate ratio (IRR).

### 2.5. Statistical Analysis

For each objective, we conducted a meta-analysis using the inverse variance random effects method. Heterogeneity was assessed by calculating I^2^. In this regard, I^2^ < 50% indicates low heterogeneity, 50–75% moderate heterogeneity, and >75% great heterogeneity [19]. As established by protocol, the detection of great heterogeneity implied that the meta-analysis or weighted mean could be biased, and consequently, that no absolute certainty could be concluded. In such cases, the causes of this great heterogeneity were analyzed. The Begg and Egger tests were used to evaluate publication bias. Due to the risk of multiple comparisons, the results of the secondary objectives were considered exclusively exploratory or generators of hypotheses. The analyses were repeated to study the influence of desert dust with each day of lag (lag 1, lag 2, lag 3, lag 4, and lag 5) after the dust episode. A weighted mean was considered to be meaningless when there were fewer than 3 articles for analysis. For this study, the results provided by the articles in the form of odds ratio, relative risk, or IRR were analyzed jointly, and the weighted mean was given as IRR with the corresponding 95% confidence interval (95% CI). The result was provided based on increments of 10 µg/m^3^ of PM_10_-dust, and a statistically significant association was considered to be present for *p* < 0.05. All the statistical analyses were carried out using the STATAIC 15.1 package (StataCorp, College Station, Lakeway, TX, USA).

## 3. Results

### 3.1. Systematic Selection of Studies

**Figure 2:**
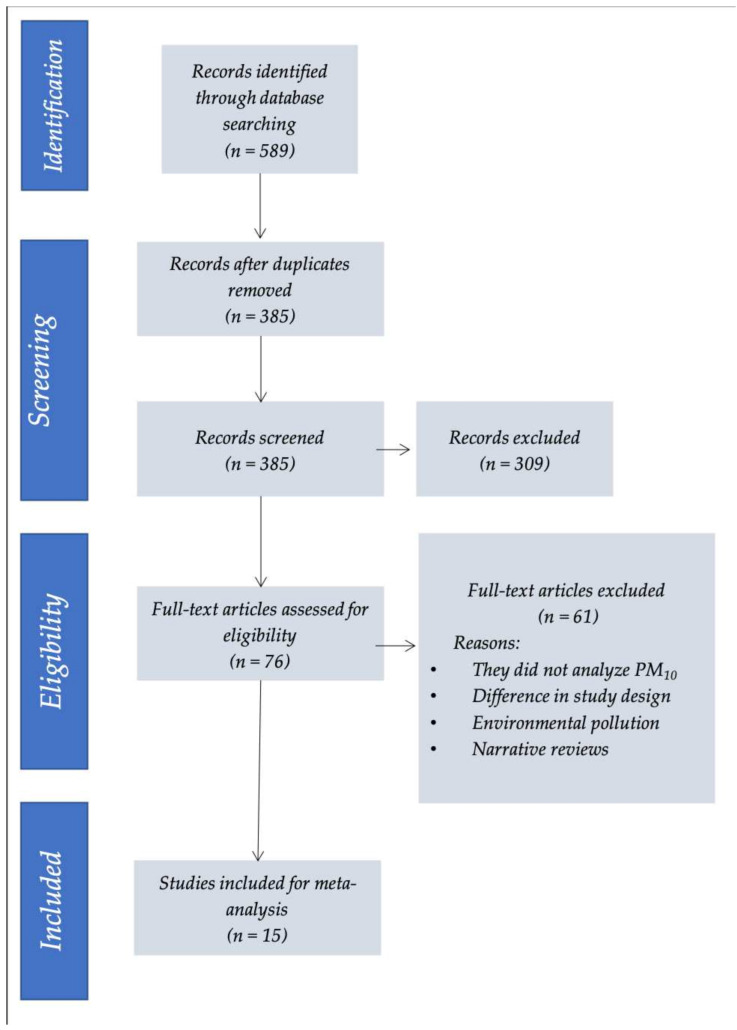
Selection of studies (PRISMA criteria).

Figure 2 shows the study flowchart according to the PRISMA criteria. A total of 589 articles were identified, of which 513 were excluded and 76 were subjected to review. Of these publications, 61 were excluded, and only 15 were found to meet all the inclusion criteria for evaluating the impact of desert dust on the incidence of cardiovascular mortality, ACS, and heart failure. The selected studies are shown in Table 1.

### 3.2. Cardiovascular Mortality

**Figure 3:**
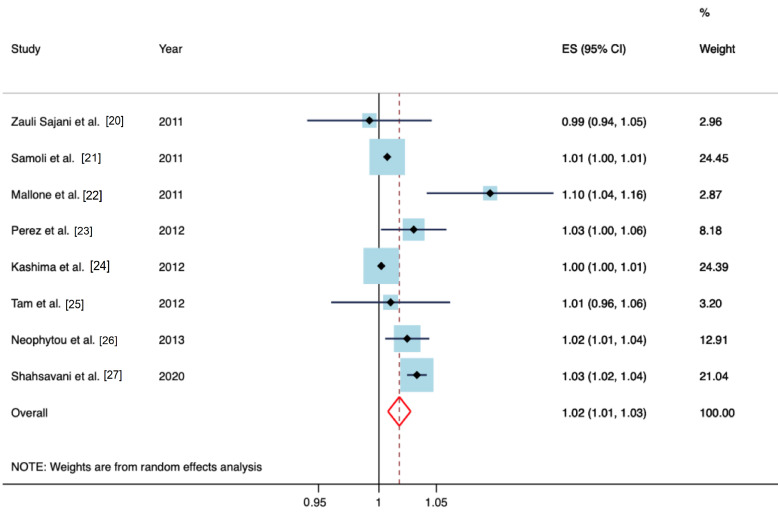
Meta-analysis (forest plot) of the influence of desert dust on cardiovascular mortality (lag 0 = on the same day). IRR: Incidence rate ratio.

Of the selected studies, 8 evaluated the impact of PM_10_-dust during desert dust episodes on cardiovascular mortality [20,21,22,23,24,25,26,27], comprising a total of 477,771 events. For day-lag 0 of the event, the impact was IRR = 1.018 (95% CI 1.008–1.027); *p* < 0.001, which represents almost a 2% daily increase in cardiovascular mortality for every 10µg/m^3^ increase in PM_10_-dust; I^2^ = 49.54% (Figure 3). The Begg and Egger tests yielded *p* = 0.71 and *p* = 0.14, respectively.

For lag 1, the impact of PM_10_-dust was IRR = 1.005 (95% CI 1.001–1.009); *p* = 0.022; I^2^ = 34.59%. The Begg and Egger tests yielded *p* = 1 and *p* = 0.44, respectively. Lastly, regarding the combined effects of exposure on the same day of the event and one day later (lag 0–1), the impact of PM_10_-dust was IRR = 1.015 (95% CI 1.003–1.028); *p* = 0.014; I^2^ = 0%. The Begg and Egger tests yielded *p* = 0.31 and *p* = 0.33, respectively (Table 2).

For lag 2 in relation to cardiovascular mortality, the impact of PM_10_-dust was IRR = 1.003 (95%CI 1–1.005); *p* = 0.04; I^2^ = 3.34%. The Begg and Egger tests yielded *p* = 0.46 and *p* = 0.52, respectively. For lag 0–5 in relation to cardiovascular mortality, the impact of PM_10_-dust was IRR = 1.023 (95%CI 0.999–1.047); *p* = 0.053; I^2^ =0%. The Begg and Egger tests yielded *p* = 0.73 and *p* = 0.88, respectively (Table 2). Fewer than three studies contributed information on the influence of PM_10_-dust in relation to lag 3, lag 4, lag 5, and lag 2–5. Therefore, no weighted means were calculated for these days.

### 3.3. ACS

Of the selected studies, only 8 provided information on the impact of PM_10_-dust on the incidence of ACS [14,24,25,28,29,30,31,32], comprising a total of 128,633 events. For lag 0 in relation to ACS, the impact of PM_10_-dust was IRR = 1.002 (95% CI 0.999–1.004); *p* = 0.144; I^2^ = 0%. The Begg and Egger tests yielded *p* = 0.67 and *p* = 0.11, respectively. For lag 1 in relation to ACS, the impact of PM_10_-dust was IRR = 1.004 (95% CI 0.999–1.009); *p* = 0.071; I^2^ = 13.39%. The Begg and Egger tests yielded *p* = 0.22 and *p* = 0.13, respectively. For lag 2 in relation to ACS, the impact of PM_10_-dust was IRR = 1.001 (95% CI 0.998–1.005); *p* = 0.449; I^2^ = 15.62%. The Begg and Egger tests yielded *p* = 0.22 and *p* = 0.59, respectively. For lag 0–1 in relation to ACS, the impact of PM_10_-dust was IRR = 1.003 (95% CI 1.001–1.006); *p* = 0.006; I^2^ = 0%. The Begg and Egger tests yielded *p* = 0.73 and *p* = 0.46, respectively. All the meta-analyses performed for different days can be seen in Table 2. Fewer than three studies reported information on the influence of PM_10_-dust in relation to lag 3, lag 4, lag 5, and lag 2–5. Therefore, no weighted means were calculated for these days.

**Figure 4:**
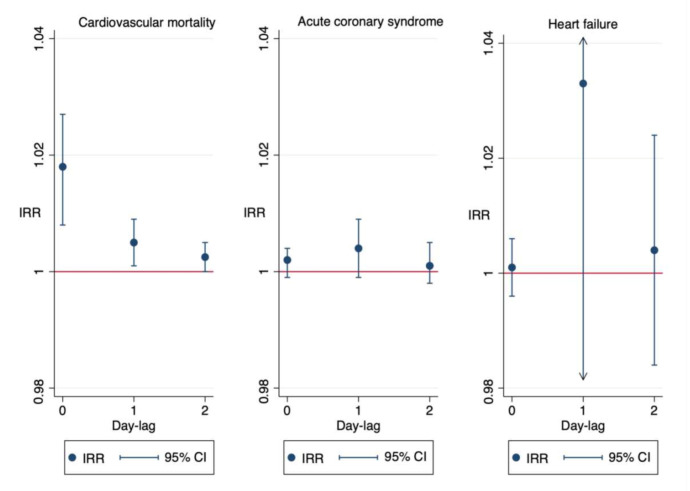
Meta-analyses performed for each objective and for each lag day from the day of exposure to desert dust until a lag of 2 days (lag 0, lag 1, and lag 2).

Figure 4 graphically displays the impact of PM_10_-dust in relation to lag 0, lag 1, and lag 2 for the different cardiovascular events studied.

### 3.4. Heart Failure

Of the selected studies, four provided information on the impact of PM_10_-dust on the incidence of admissions due to heart failure [25,29,30,33], comprising a total of 84,192 events. For lag 0 in relation to heart failure, the impact of PM10-dust was IRR = 1.001 (95% CI 0.996–1.006); *p* = 0.67; I^2^ = 0%. The Begg and Egger tests yielded *p* = 0.73 and *p* = 0.94, respectively. For lag 1 in relation to heart failure, the impact of PM_10_-dust was IRR = 1.033 (95% CI 0.977–1.091); *p* = 0.253; I^2^ = 0%. The Begg and Egger tests yielded *p* = 0.29 and *p* = 0.06, respectively. For lag 2 in relation to heart failure, the impact of PM_10_-dust was IRR = 1.004 (95% CI 0.984–1.024); *p* = 0.698; I^2^ = 0%. The Begg and Egger tests yielded *p* = 0.30 and *p* = 0.71, respectively (Table 2). Fewer than three studies contributed information on the influence of PM_10_-dust in relation to lag 3, lag 4, lag 5, lag 0–1, lag 0–5, and lag 2–5. Therefore, no weighted means were calculated for these days.

## 4. Discussion

Based on studies of time series and cross-case designs with strict inclusion criteria and almost 700,000 events, the present meta-analysis provides strong evidence of an association between desert dust and cardiovascular mortality. This association was seen to be stronger on the same day of exposure to dust and weaker over the following days. To the best of our knowledge, this is the first meta-analysis on the effect of desert dust on cardiovascular events.

**Figure 5:**
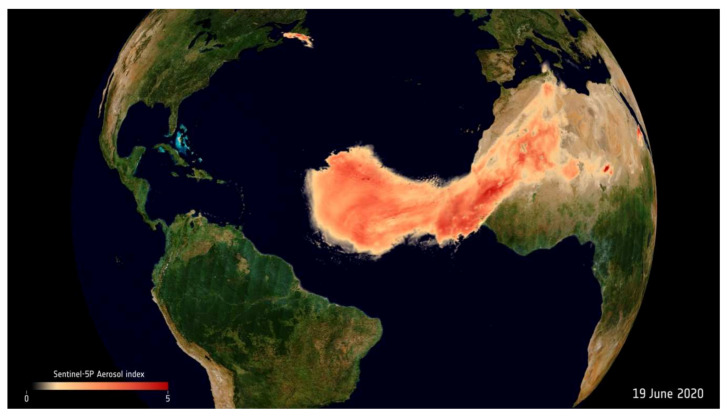
Data from the Copernicus Sentinel satellites and ESA’s Aeolus satellite show the extent of this year’s summer dust plume, dubbed “Godzilla,” on its journey across the Atlantic.

Our results are of utmost importance. Every year, especially in summer, Saharan dust travels across the Atlantic and reaches several parts of the world. In June 2020, a tremendous plume of dust from North Africa was detected approaching the USA and other countries (Figure 5 and Supplementary Materials Video S1). The intensity and extent of the plume was so great that it blanketed the Caribbean Sea and darkened skies in several states in the USA [34].

Different studies conducted individually in different regions have evaluated the relationship between desert dust and ACS [14,24,25,28,29,30,31,32] and heart failure [25,29,30,33]. In our opinion, the number of studies made is clearly insufficient to be able to draw conclusions. Moreover, many of the studies have been carried out in regions where local and regional pollution contributes far more than desert dust to the PM_10_ levels, i.e., PM_10_-pollution-combustion > PM_10_-dust. 

**Figure 6:**
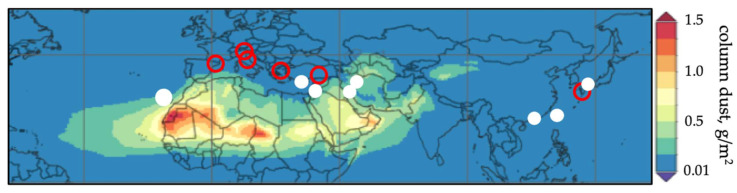
Distribution of the regions in which the studies on dust and cardiovascular disorders have been carried out based on the systematic review. Note the regions (white dot) in which the PM_10_ levels during the dust episodes are high, i.e., two or more times higher than those recorded in the absence of desert dust (from west to east): Tenerife—Spain, Nicosia—Cyprus, BéerSheva—Israel, Ahvaz—Iran, Teheran—Iran, Hong Kong—China, Taipei—Taiwan, and Fukuoka—Japan. Note the regions (red circle) in which the PM_10_ levels during the dust episodes do not increase significantly, i.e., without reaching two times those recorded in the absence of desert dust (from west to east): Barcelona—Spain, Brescia—Italy, Emilia Romagna—Italy, Athens—Greece, Gaziantep—Turkey, and western Japan. Column dust is shown according to MERRA-2 model.

Table 1 shows the PM_10_ values during the dust and non-dust episodes (PM_10_ of component dominated by local and regional pollution) in each study. It also shows the PM_10_ during dust episodes and PM_10_ non-dust episodes ratio as a measure of the “increase” in PM_10_ during the desert dust events. It can be seen that the studies carried out in southern Europe (Greece, Italy, and continental Spain) and Turkey reflect a truly modest increment of between 1.0–1.4, i.e., PM_10_ did not increase significantly during the desert dust episodes, since the mean increment was 37 to 48µg/m^3^. In Brescia (northern Italy), the PM_10_ even decreased during the desert dust events (42 to 38µg/m^3^). These study zones are shown in Figure 6 (red circle).

In the study carried out in Barcelona (Spain), PM_10_ reached ~39µg/m^3^ during the Saharan episodes, of which only 16 µg/m^3^ corresponded to desert dust—a load similar to that recorded in other zones of southern Europe [23]. In addition, the climatic conditions under which these desert dust events take place often favor the accumulation of local [35] and regional pollutants (thermal inversions secondary to hot air advection at high altitude) [36,37], which can also contribute to the adverse effects on health.

In contrast, in the studies carried out in Asia (Japan, Taiwan and China), the Middle East (Iran and Israel), northern Africa (Tenerife), and Cyprus, the PM_10_ values experienced an important increase during the dust episodes, with a PM_10_ during dust episodes and PM_10_ non-dust episodes ratio of between 2–53, and PM_10_ increments from 54 µg/m^3^ to 157µg/m^3^ on average (2650 µg/m^3^ in Israel). These studies were made in the so-called Dust Belt (Figure 6, white dot). In our opinion, these are the regions where further research is needed. With regard to the studies addressing heart failure and ACS, only two [25,33] (out of a total of four) and three [14,24,28] (out of a total of five) were carried out in that region, respectively.

Desert dust has a significant impact on air quality not only in areas close to the source points or regions, but also over areas up to a few thousand kilometers away [4].

### Limitations

Some limitations of our analysis need to be acknowledged. First, we found a moderate degree of inconsistency. Due to the paucity of studies available, we could not perform subgroup analysis or meta-regression that could shed light in the presence of inconsistency. Second, we reported the estimated effect as IRR. Data extraction of the different studies included the estimated effect based on the odds ratio, relative risks, and IRR. The measurements of the odds ratio and relative risks would be almost equivalent when risks are very low [38]. Third, although this meta-analysis was conceived to increase statistical power and improve precision, we cannot rule out a lack of statistical power, especially in some endpoints with few involved studies or others with some degree of inconsistency. Fourth, the effects of desert dust on health depend on the trajectory and distance between the desert dust storms and the human populations [8]. Finally, the studies included in the present meta-analysis may have been influenced by geographical bias, since 67% of them were made outside the Dust Belt—a fact that may condition the absence of statistical significance referred to cardiovascular events in the form of ACS and heart failure. Caution should therefore be exercised when interpreting the pooled risk estimates and highlights the need for more data to increase the certainty of these risk estimates [39].

## 5. Conclusions

This is the first meta-analysis that has demonstrated that exposure to desert dust results in a 2% increase (for every 10µg/m^3^ of PM_10_-dust) in cardiovascular mortality risk as assessed on the same day of exposure. Desert dust also increases the cardiovascular mortality risk as assessed 1 and 2 days after exposure, though, in this case, the effect is less important. The results of this study may help to alert the most vulnerable populations to adopt adequate measures during desert dust episodes.

## Figures and Tables

**Table 1:** Studies on the impact of desert dust on cardiovascular events identified in the systematic review for meta-analysis. The fraction of PM_10_ linked to dust (DE) and no dust (no-DE) event is shown.

Authors	Region	Country	Design	CE	PM_10_ DE	PM_10_ no-DE	R	Period	Risk of Bias
Zauli, 2011 [20]	Emilia Romagna	Italy	CC	CM	38	42	**0.9**	2002–2006	Low
Samoli, 2011 [21]	Athens	Greece	TS	CM	47	39	**1.2**	2001–2006	Low
Malone, 2011 [22]	Rome	Italy	CC	CM	52	37	**1.4**	2001–2004	Low
Perez, 2012 [23]	Barcelona	Spain	TS	CM	39	NS		2003–2007	Moderate
Kashima, 2012 [24]	5 cities	Japan	TS	CM/ACS	184	22	**8.3**	2005–2010	Low
Tam, 2012 [25]	Hong Kong	China	CC	CM/ACS/HF	134	50	**2.7**	1998–2002	Low
Neophytou, 2013 [26]	Nicosia	Cyprus	TS	CM	116	53	**2.2**	2004–2007	Low
Shahsavani, 2020 [27]	Ahvaz	Iran	CC	CM	266	101	**2.6**	2015–2017	Low
Shahsavani, 2020 [27]	Tehran	Iran	CC	CM	192	82	**2.3**	2015–2017	Low
Dominguez-Rodriguez, 2020 [14]	Tenerife	Spain	CC	ACS	92	16	**5.6**	2014–2017	Low
Vodonos, 2015 [28]	Béer-Sheva	Israel	CC	ACS	2650	50	**53**	2001–2010	Low
Vaduganathan, 2016 [29]	Brescia	Italy	TS	ACS/HF	NS	NS		2004–2007	Moderate
Al, 2018 [30]	Gaziantep	Turkey	TS	ACS/HF	74	72	**1.0**	2009–2014	Low
Zhang, 2016 [31]	Beijing	China	CC	ACS	NS	NS		2014–2014	Moderate
Matsukawa, 2014 [32]	Fukuoka	Japan	CC	ACS	55	29	**1.9**	2003–2010	Low
Yang, 2009 [33]	Taipei	Taiwan	CC	CI	112	55	**2.0**	1996–2001	Low

The design specifies whether the study was cross-case (CC) or time series (TS). All studies were based on Poisson regression. Under cardiovascular event (CE), it is specified whether cardiovascular mortality (CM), heart failure (HF), and/or acute coronary syndrome (ACS) was studied. For each study, PM_10_ (in µg/m^3^) during the dust events (DE) and in the absence of dust events (no-DE) is shown, together with the PM_10_ DE/no-DE ratio (R). NS: Data not supplied in the study. The column R marks in boldface the studies in which the increase in PM_10_ levels during the dust episodes versus the non-dust episodes (no-DE) proved relevant (R ≥ 2).

**Table 2:** Influence of desert dust on cardiovascular mortality, acute coronary syndrome and heart failure for the different lag days. Lag 0 = same day; lag 1= lag of one day; lag 2 = lag of 2 days; lag 3 = lag of 3 days; lag 4 = lag of 4 days; lag 0–1 = lag of 0–1 day; lag 0–5 = lag of 0–5 days.

Objective	Incidence Rate Ratio	*p*-Value	I^2^	Egger (*p*-Value)	Begg (*p*-Value)
Cardiovascular mortality					
Lag 0	1.018 (95%CI 1.008–1.027)	<0.001	49.54%	0.14	0.71
Lag 1	1.005 (95%CI 1.001–1.009)	0.022	34.59%	0.44	1.00
Lag 2	1.003 (95%CI 1.000–1.005)	0.040	3.34%	0.52	0.46
Lag 0–1	1.015 (95%CI 1.003–1.028)	0.014	0.00%	0.33	0.31
Lag 0–5	1.023 (95%CI 0.999–1.047)	0.053	0.00%	0.88	0.73
Acute coronary syndrome					
Lag 0	1.002 (95%CI 0.999–1.004)	0.144	0.00%	0.11	0.67
Lag 1	1.004 (95%CI 0.999–1.009)	0.071	13.39%	0.13	0.22
Lag 2	1.001 (95%CI 0.998–1.005)	0.449	15.62%	0.59	0.22
Lag 3	0.986 (95%CI 0.949–1.028)	0.507	6.24%	0.14	0.60
Lag 4	1.025 (95%CI 0.902–1.164)	0.706	43.07%	0.34	0.29
Lag 0–1	1.003 (95%CI 1.001–1.006)	0.006	0.00%	0.46	0.73
Heart failure					
Lag 0	1.001 (95%CI 0.996–1.006)	0.670	0.00%	0.94	0.74
Lag 1	1.033 (95%CI 0.977–1.091)	0.253	0.00%	0.06	0.29
Lag 2	1.004 (95%CI 0.984–1.024)	0.698	0.00%	0.71	0.30

## Data Availability

Not applicable.

## Supplementary Materials

The following are available online at https://www.mdpi.com/2077-0383/10/4/727/s1, Figure S1: Search strategies, Video S1: Supplementary video.
