# Supplementary figures and images for: Impact of Desert Dust Events on the Cardiovascular Disease: A Systematic Review and Meta-Analysis
Source: J Clin Med. 2021 Feb 12;10(4):727. doi: 10.3390/jcm10040727 (PMC7918944; doi:10.3390/jcm10040727)

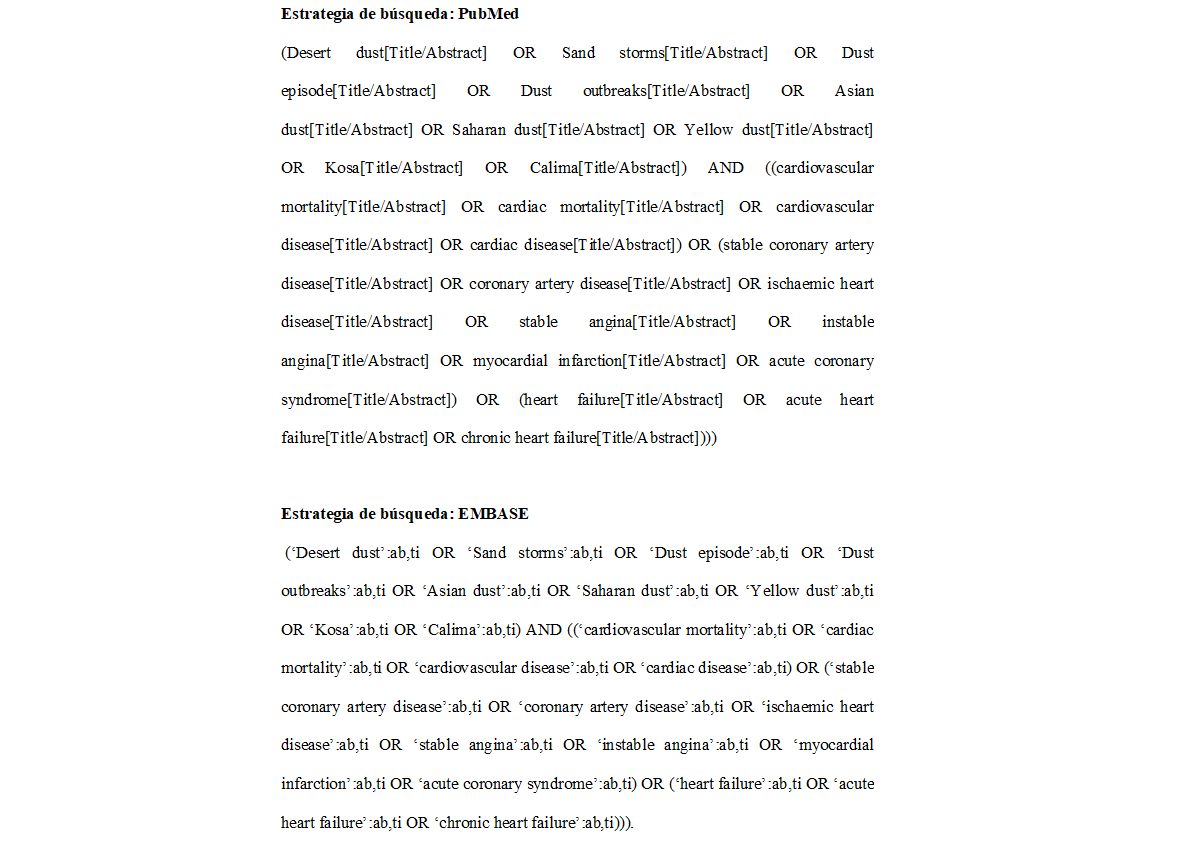

Supplement: Supplementary file 1 [file jcm-10-00727-s001.zip › SupplementayFigure-1.png]
